# An Unusual Presentation of Massive Asymptomatic Urinary Retention in a 63-Year-Old Male With Iron Deficiency Anaemia

**DOI:** 10.7759/cureus.86808

**Published:** 2025-06-26

**Authors:** Daniel Akintelure, Simon Akintelure, Ganiyat A Adeshina

**Affiliations:** 1 Urology, Royal Gwent Hospital, Newport, GBR; 2 Urology, All Saints University School of Medicine, Roseau, DMA; 3 Pharmacology and Therapeutics, University of Lagos, Lagos, NGA

**Keywords:** chronic urinary retention, iron deficiency anaemia, prostatic hyperplasia, transurethral resection of the prostate (turp), urinary catheter

## Abstract

Chronic urinary retention (CUR) is typically symptomatic, but rare cases exist where patients present without symptoms. We report on a 63-year-old man with a huge, distended bladder, holding approximately 15 litres of urine at the time of examination. Despite the bladder’s size, the patient was completely asymptomatic, with preserved renal function. Management included catheterization with intermittent clamping to prevent rapid decompression, and initiation of tamsulosin and finasteride for suspected benign prostatic enlargement (BPE), with plans for transurethral resection of the prostate (TURP) as definitive management. Even without symptoms, CUR can exist and risks serious complications if not identified and managed early.

## Introduction

Urinary retention is the inability to completely empty the bladder and is typically classified as either acute or chronic. Chronic urinary retention (CUR) refers to a prolonged condition in which the bladder fails to empty adequately over time, often without the discomfort or urgency that characterizes acute retention [1]. CUR is further categorized into two forms based on intravesical pressure and the impact on the upper urinary tract: high-pressure and low-pressure CUR [2,3].

High-pressure CUR occurs when prolonged bladder outlet obstruction leads to elevated intravesical pressures, eventually causing backpressure effects such as hydronephrosis and renal dysfunction [2]. This form is particularly concerning due to the potential for irreversible renal damage if left untreated. In contrast, low-pressure CUR is associated with a compliant, distensible bladder that accommodates large urine volumes while maintaining low intravesical pressure, often sparing the kidneys [3]. Patients with low-pressure CUR can remain asymptomatic for extended periods, making diagnosis challenging and often incidental. This bladder adaptation is thought to be the result of gradual detrusor stretching, reduced sensation, and increased compliance over time [1-3].

CUR is commonly caused by benign prostatic enlargement (BPE), which is the leading aetiology in older men. Other causes include urethral strictures, pelvic masses, neurogenic bladder, and detrusor underactivity resulting from neurological conditions such as diabetes mellitus, Parkinson’s disease, multiple sclerosis, or spinal cord injuries [4-6]. In detrusor underactivity, the bladder muscle fails to contract effectively, leading to incomplete bladder emptying without necessarily causing obstructive symptoms.

The diagnosis of CUR is typically established when the post-void residual (PVR) urine volume exceeds 300 mL, measured via bladder ultrasound or catheterization [1,7]. CUR may also present with secondary complications such as bladder wall thickening, upper tract dilation, urinary tract infections, or impaired renal function. However, no universally accepted PVR threshold exists, and clinical context remains essential in guiding diagnosis and management.

While precise prevalence data are limited due to underdiagnosis and asymptomatic presentations, studies suggest that up to 6% of men over the age of 60 may have undetected CUR, with the incidence increasing with age and the presence of risk factors [6,8]. Importantly, the absence of lower urinary tract symptoms (LUTS) does not preclude significant urinary retention or obstructive pathology.

We present a rare and remarkable case of massive asymptomatic CUR, incidentally discovered during evaluation for iron deficiency anaemia in a 63-year-old male. Imaging revealed a bladder containing approximately 15 litres of urine, yet the patient exhibited no LUTS and had preserved renal function at presentation. This case emphasizes the importance of maintaining a high index of suspicion for urinary retention during the investigation of anaemia, particularly in elderly men, and demonstrates the bladder’s extraordinary capacity for silent distension in CUR.

## Case presentation

A 63-year-old married male, working as a social worker, was referred to the gastroenterology department for evaluation of unexplained iron deficiency anaemia (IDA). His primary symptoms included lethargy and fatigue. He denied any gastrointestinal symptoms, bleeding episodes, weight loss, or systemic signs such as fever or back pain. There was no relevant family history, and he was not taking any medications at the time of presentation.

During abdominal examination, a grossly distended bladder was palpable. Digital rectal examination (DRE) revealed a smooth, enlarged prostate consistent with benign findings. Despite the significant urinary retention and upper tract changes, the patient remained asymptomatic with no LUTS such as urgency, frequency, nocturia, or incontinence.

Initial laboratory investigations, as seen in Table 1, revealed a haemoglobin level of 105 g/L, consistent with IDA. A complete blood count (CBC), mean cell volume (MCV), ferritin levels, and thyroid function tests were otherwise unremarkable. In accordance with the British Society of Gastroenterology (BSG) guidelines, which recommend endoscopic evaluation in all adult males and postmenopausal females with unexplained IDA to exclude gastrointestinal malignancy, the patient underwent upper gastrointestinal endoscopy and colonoscopy [9]. The gastroscopy demonstrated mild reflux esophagitis, while the colonoscopy findings were unremarkable.

**Table 1:** Values of laboratory examinations for investigation of iron deficiency anaemia.

Parameter	Result	Reference Range
Haemoglobin (g/L)	105	130-180
Mean Cell Volume (fL)	96	80-100
Folate (ug/L)	18.1	>3
Ferritin (ug/L)	25	15-300
Vitamin B12 (ng/L)	795	180-900
Thyroid Stimulating Hormone (mU/L)	2.64	0.55-4.78
Free T4 (pmol/L)	15.7	11.5-22.7
White blood cell (x10^9/L)	4.3	4.0-11.0
Platelets (x10^9/L)	282	150-400
Red blood cell (x10^12/L)	3.32	4.50-6.00
Haematocrit (L/L)	0.32	0.40-0.52
Mean Cell Haemoglobin (pg)	31.6	27.0-33.0
Red Cell Distribution Width (%)	13.5	11.0-14.8
Neutrophil (x10^9/L)	2.6	1.7-7.5
Lymphocyte (x10^9/L)	0.9	1.0-4.5
Monocyte (x10^9/L)	0.6	0.2-0.8
Eosinophil (x10^9/L)	0.2	0.0-0.4
Basophil (x10^9/L)	0	0.0-0.1

**Figure 1:**
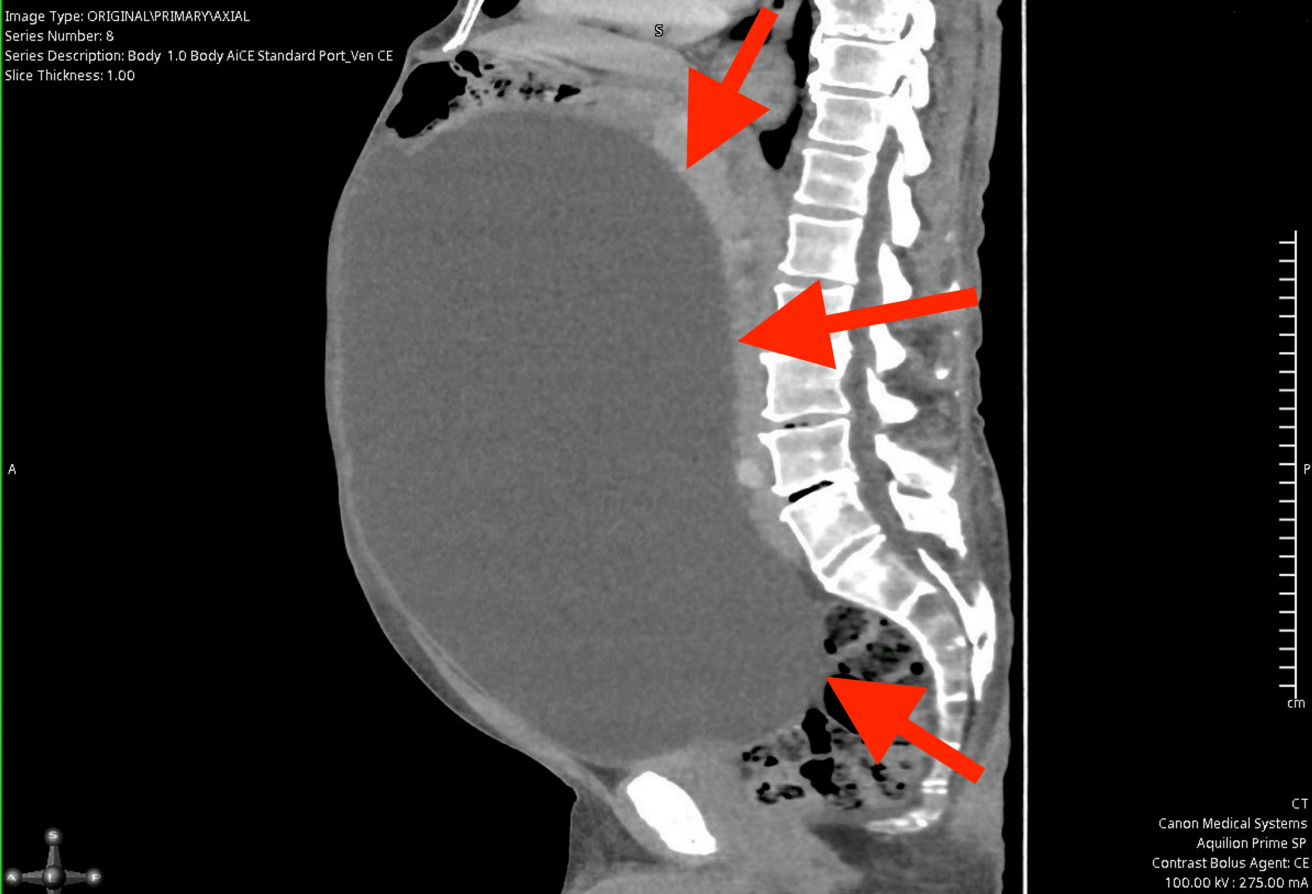
Computed Tomography scan of abdomen and pelvis illustrating largely distended bladder (sagittal view), the red arrows indicate the distended bladder wall.

**Figure 2:**
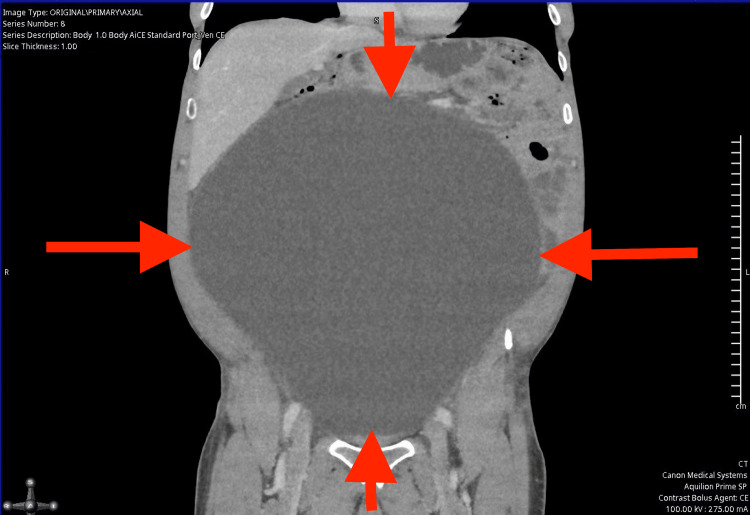
Computed Tomography scan of abdomen and pelvis illustrating largely distended bladder (coronal view), the red arrows indicate the distended bladder wall.

**Figure 3:**
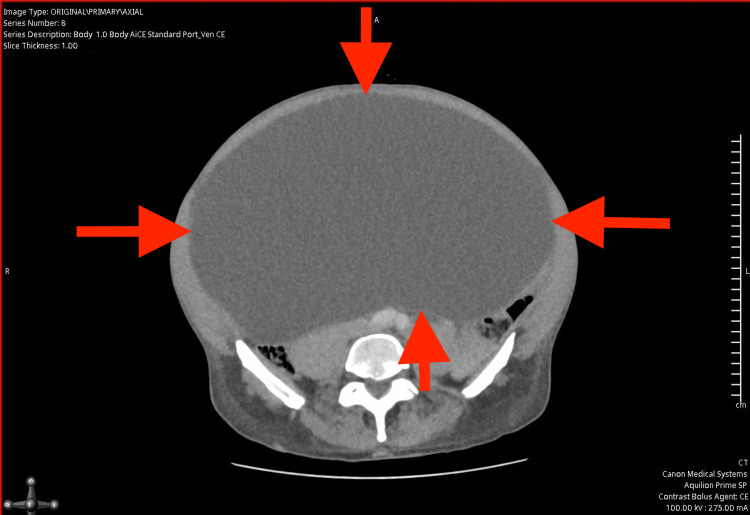
Computed Tomography scan of abdomen and pelvis illustrating largely distended bladder (axial view), the red arrows indicate the distended bladder wall.

**Figure 4:**
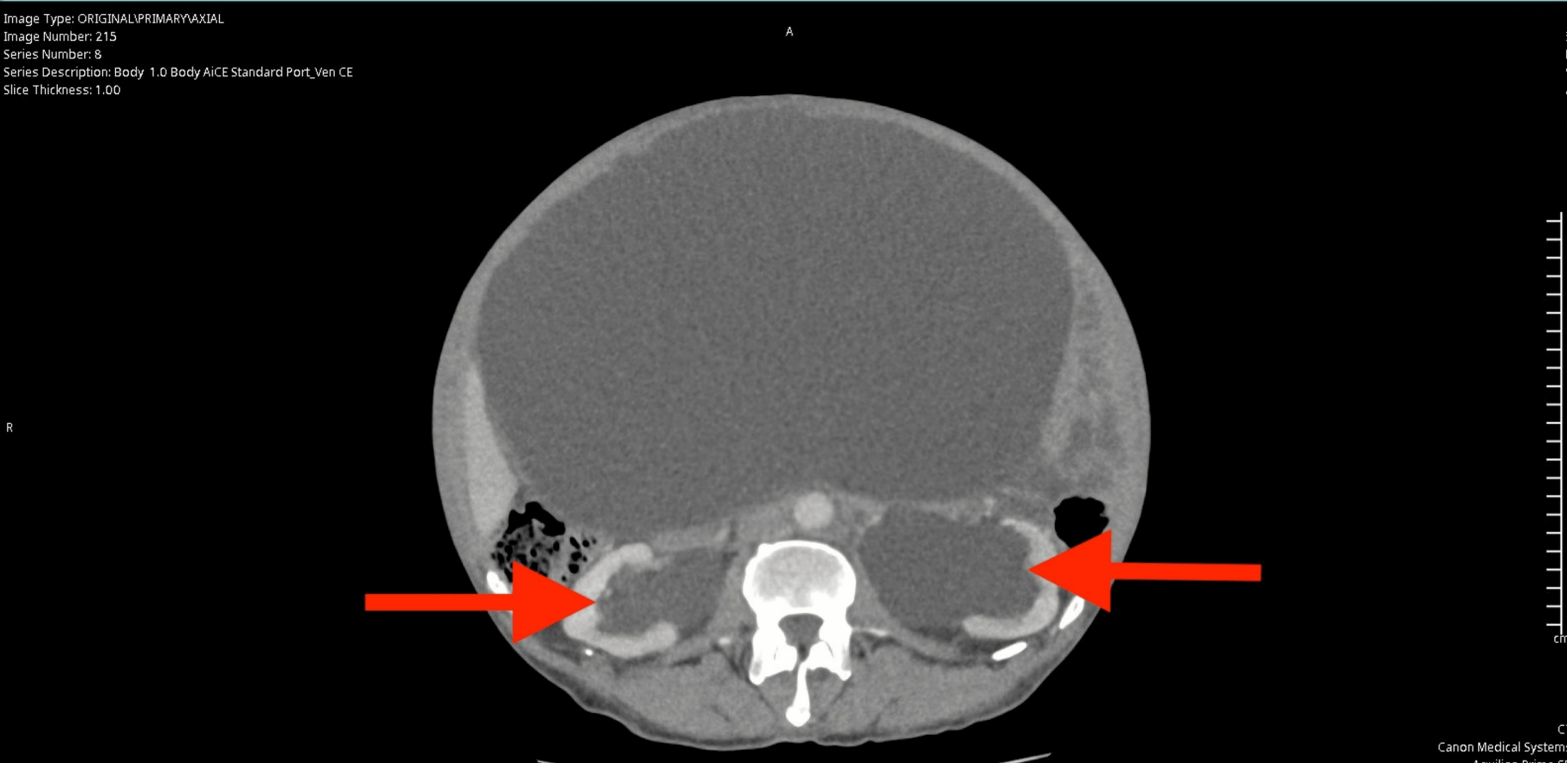
Computed Tomography scan of abdomen and pelvis illustrating largely distended bladder (axial view), the red arrows indicate the associated bilateral hydronephrosis.

Due to the absence of a gastrointestinal source of anaemia, a computed tomography (CT) scan of the abdomen and pelvis was performed to further investigate the cause. The CT (Figures 1-4) revealed a massively distended urinary bladder containing approximately 15 litres of urine. Additionally, it showed associated bilateral hydroureter and hydronephrosis, suggesting chronic bladder outlet obstruction. The prostate was measured at approximately 60 cc in volume - significantly enlarged compared to the normal adult prostate volume of 20-30 cc [10].

He was referred to the urology team for further management. A urinary catheter (18 French) was inserted carefully to initiate bladder drainage by the urology team. Intermittent clamping was performed hourly to ensure gradual decompression, adhering to best practices designed to minimise the risks associated with sudden decompression. Rapid emptying of a chronically over-distended bladder can lead to complications such as haematuria, hypotension, and post-obstructive diuresis due to abrupt changes in intravesical pressure and circulatory dynamics. These risks have been documented in multiple case reports and clinical observations [11,12]. After insertion of the urinary catheter, the bladder drained 13 litres of urine into the catheter bag.

Initial renal function tests, as seen in Table 2, demonstrated a creatinine level of 114 µmol/L and an estimated glomerular filtration rate (eGFR) of 56 mL/min/1.73 m². Electrolyte values were within normal range. Post-catheterisation, his creatinine decreased slightly to 102 µmol/L with a corresponding increase in eGFR to 64 mL/min/1.73 m² as seen in Table 3, indicating a mild improvement in renal function, likely due to relief of obstructive uropathy.

**Table 2:** Values of laboratory examinations of renal function and prostate specific antigen.

Parameter	Result	Reference Range
Creatinine (umol/L)	114	58-110
Sodium (mmol/L)	142	133-146
Potassium (mmol/L)	4.5	3.5-5.3
Estimated Glomerular Filtration Rate (ml/min/1.73m2)	56	>90
Prostate Specific Antigen (ug/L)	0.3	<4.6

**Table 3:** Values of laboratory examinations of renal function following catheterisation.

Parameter	Result	Reference Range
Creatinine (umol/L)	102	58-110
Sodium (mmol/L)	139	133-146
Potassium (mmol/L)	3.9	3.5-5.3
Estimated Glomerular Filtration Rate (ml/min/1.73m2)	64	>90
Urea (mmol/L)	6.5	2.5-7.8

Based on CT and DRE findings, a diagnosis of BPE was made, which was identified as the likely cause of the chronic urinary retention. The patient was started on medical therapy including tamsulosin 400 micrograms once daily and finasteride 5 mg once daily. Tamsulosin was initiated to reduce bladder outlet resistance by relaxing smooth muscle in the prostate and bladder neck. The desired aim was to facilitate spontaneous voiding during the planned trial without catheter (TWOC). It was believed that medical therapy would optimise the chance of successful voiding after catheter removal and may prevent future episodes of retention.

The patient was discharged with an indwelling catheter and instructions for intermittent self-catheterisation, which he performed three times daily. He returned for a follow-up urology clinic appointment one week later. At that visit, no additional laboratory investigations were performed. He reported compliance with medical therapy and self-catheterisation. It was further confirmed that he was diagnosed with BPE based on his previous findings on CT imaging and DRE. Based on his clinical findings and persistent retention, he was listed for an outpatient transurethral resection of the prostate (TURP) as definitive treatment.

## Discussion

CUR is a urological condition defined by a persistent inability to fully empty the bladder, typically associated with a PVR urine volume exceeding 300 mL over a prolonged period [2]. The true prevalence of CUR is difficult to determine due to its often insidious and asymptomatic presentation. However, estimates suggest that approximately 10-15% of men over the age of 70 years may have some degree of CUR, with rates rising sharply in those with concomitant BPE [13]. The condition can be categorised into two subtypes: low-pressure CUR, characterised by painless retention and compliant bladder, and high-pressure CUR, where increased detrusor pressures lead to upper tract damage such as hydroureter, hydronephrosis, and renal dysfunction [3].

CUR is often the result of bladder outlet obstruction (BOO), with BPE being the most prevalent etiology in ageing men. Other causes include urethral stricture, detrusor underactivity, and neurogenic bladder. Investigations for CUR typically begin with a focused history and physical examination, including a DRE to assess prostate size and consistency. Imaging studies, such as ultrasound or CT, help quantify bladder distension, prostate size, and assess for hydronephrosis. Uroflowmetry and measurement of PVR using bladder scan or catheterisation are critical in confirming the diagnosis. In some cases, urodynamic studies may be necessary to differentiate between detrusor underactivity and outlet obstruction [14].

Management of CUR depends on the underlying etiology and patient factors. In men with BPE-related CUR, initial management includes bladder decompression via catheterisation, followed by pharmacotherapy with alpha-blockers and 5-alpha-reductase inhibitors. Alpha-blockers like tamsulosin act by relaxing smooth muscle in the prostate and bladder neck, thereby reducing outlet resistance. Finasteride reduces prostate volume by inhibiting the conversion of testosterone to dihydrotestosterone, leading to long-term symptomatic relief and reduced risk of progression [15,16].

In cases where medical therapy fails or is insufficient, such as persistent retention, recurrent urinary tract infections, or impaired renal function, surgical options must be considered. TURP remains the gold standard for surgical management of BPE and CUR. It offers symptomatic relief, improves urinary flow, and reduces the need for long-term catheterisation. Despite its invasive nature, TURP is generally well-tolerated and effective in relieving obstruction and restoring bladder function [17].

Bladder decompression in CUR must be approached cautiously. Though earlier dogma suggested a need for gradual decompression to avoid complications such as hematuria and hypotension, evidence has been mixed. A systematic review by Zhang et al. found no significant difference in complication rates between gradual and rapid decompression, but gradual approaches are still commonly adopted due to theoretical hemodynamic advantages [18].

A potential complication post-decompression is post-obstructive diuresis, seen in up to 52% of patients, particularly when significant bladder overdistension and renal dysfunction have occurred [19]. This diuresis may lead to hypovolemia and electrolyte imbalances if not properly monitored. Clinical vigilance with input/output monitoring and fluid/electrolyte management is essential in the immediate post-decompression period [20].

## Conclusions

In conclusion, this case highlights a rare and atypical presentation of CUR, where a 63-year-old male remained entirely asymptomatic despite a massively distended bladder containing approximately 15 litres of urine and significant bilateral hydroureter and hydronephrosis. The incidental discovery during evaluation for unexplained iron deficiency anaemia underscores the importance of a holistic and guideline-driven diagnostic approach, particularly in men and postmenopausal women, where malignancy and non-gastrointestinal sources must be excluded.

While BPE is a common urological condition in ageing men, this case illustrates an unusual scenario where it caused severe bladder outlet obstruction without the typical lower urinary tract symptoms. The patient’s management involved cautious bladder decompression, pharmacological intervention, and eventual listing for TURP, reflecting current best practices for CUR management.

This case reinforces the clinical value of considering urological causes in patients with unexplained IDA and demonstrates the critical role of cross-specialty collaboration. Furthermore, it emphasises that even advanced presentations of CUR may be silent, necessitating a high index of suspicion and comprehensive investigation.
